# Human Metapneumovirus Small Hydrophobic Protein Inhibits Interferon Induction in Plasmacytoid Dendritic Cells

**DOI:** 10.3390/v10060278

**Published:** 2018-05-23

**Authors:** Xiaoyong Bao, Deepthi Kolli, Dana Esham, Thangam S. Velayutham, Antonella Casola

**Affiliations:** 1 Department of Pediatrics, The University of Texas Medical Branch, Galveston, TX 77555, USA; 2 Atara Biotherapeutics, Oak Park, CA 94080, USA; dkolli@atarabio.com; 3 Adena Health System, Chillicothe, OH 45601, USA; desham@adena.org; 4 Department of Pathology, The University of Texas Medical Branch, Galveston, TX 77555, USA; thvelayu@utmb.edu; 5 Department of Microbiology & Immunology, The University of Texas Medical Branch, Galveston, TX 77555, USA; 6 Sealy Center for Vaccine Development, The University of Texas Medical Branch, Galveston, TX 77555, USA

**Keywords:** hMPV, SH protein, plasmacytoid dendritic cells, type I IFN

## Abstract

Human metapneumovirus (hMPV), a leading cause of respiratory tract infections in infants, encodes a small hydrophobic (SH) protein of unknown function. Here we show that infection of plasmacytoid dendritic cells (pDCs) with a recombinant virus lacking SH expression (rhMPV-ΔSH) enhanced the secretion of type I interferons (IFNs), which required TLR7 and MyD88 expression. HMPV SH protein inhibited TLR7/MyD88/TRAF6 signaling leading to IFN gene transcription, identifying a novel mechanism by which paramyxovirus SH proteins modulate innate immune responses.

## 1. Introduction

Human metapneumovirus (hMPV) is a leading cause of both upper and lower respiratory tract infections in infants, elderly and immunocompromised patients worldwide [1]. Since its identification, hMPV has been isolated from individuals of all ages with acute respiratory tract infection worldwide [2], and virtually all children older than five years show 100% serologic evidence of infection [3]. 

The hMPV small hydrophobic (SH) protein is a type II transmembrane glycoprotein [4], whose function is not well understood. Using recombinant hMPV viruses (derived from the hMPV CAN83, an A2 strain, as template), either wild type (rhMPV-WT) or lacking SH (rhMPV-ΔSH) [5,6], we have shown that hMPV SH protein regulates host immune responses in epithelial cells by modulating nuclear factor kappa-light-chain-enhancer of activated B cells (NF-κB) activation [6]. However, its function in immune cells is yet to be determined. 

One of the initial and important responses to viral infection is the rapid release of type I interferons (IFNs) [7]. Although most types of cells can secrete IFN [8,9], plasmacytoid dendritic cells (pDC) represent an important source of IFN produced upon the entry of bacterial and viral pathogens [10,11,12]. Several viruses have evolved tools to counteract IFN production by pDC. In recent investigations, we have shown that hMPV can infect human pDCs and that this infection inhibits toll-like receptors (TLR)-dependent signaling [13], similar to what has been described for the respiratory syncytial virus (RSV) and measles virus [14,15]. In this study, we found that hMPV SH protein inhibited TLR7/myeloid differentiation primary response 88 (MyD88)/TNF receptor associated factor 6 (TRAF6) signaling and suppressed IFN gene expression, identifying a novel mechanism by which hMPV SH proteins modulate innate immune responses.

## 2. Materials and Methods 

### 2.1. Virus Preparation 

Recombinant viruses derived from hMPVCAN-83, WT, or SH-deleted, were propagated and purified, as previously described [6,16]. Viral titer was determined by immunostaining in LLC-MK2 cells, as previously described [6,16]. 

### 2.2. hMPV pDC Preparation and Infection

Human pDCs were isolated from peripheral blood mononuclear cells (PBMCs, donated by young healthy donors) using a diamond plasmacytoid cell isolation kit (Miltenyi Biotec, Auburn, CA, USA), as previously described [13]. Purified pDCs (90–98% pure) were infected with recombinant hMPV, either wild type (rhMPV-WT) or lacking SH protein expression (rhMPV-ΔSH), generated as previously described [6], at a multiplicity of infection (MOI) of 3. The percentage of infected pDCs was determined by immunostaining as previously described [17]. Briefly, virus-infected cells were first fixed with Cytofix/cytoperm (Pharmingen, San Jose, CA, USA), permeabilized with Perm/wash buffer (Pharmingen) and incubated with guinea pig anti-hMPV antibody, followed by a FITC-goat anti-guinea pig antibody (Zymed, South San Francisco, CA, USA). Cells were analyzed with a FACScan flow cytometer equipped with CellQuest software (both from Becton Dickinson Immunocytometry Systems, San Jose, CA, USA) and analysis was performed in FlowJo software 10 (Treestar, CA, USA).

### 2.3. Interferon Quantification 

Interferon (IFN)-α, and -β levels in pDC cell supernatants were quantified by enzyme-linked immunosorbent assay (ELISA, PBL Biomedical Laboratories, Piscataway, NJ, USA).

### 2.4. Reporter Gene Assays

In experiments where SH, in modulating TLR-7-mediated signaling pathway, was investigated, HEK293 cells stably expressing TLR7 (Invivogen, San Diego, CA, USA), or their wild type counterpart, were transfected in triplicate with a synthetic interferon stimulated responsive element (ISRE)-driven luciferase reporter gene plasmid (controlled by interferon regulatory factors (IRFs) activation), together with an SH expression plasmid, or its empty vector using FuGene 6 (Roche, Basel, Switzerland). The next day cells were treated with 1 mM Loxoribine (TLR7 agonist, Invivogen) and harvested at 40 h post-transfection to measure luciferase activity, as previously described [6,18].

To investigate the role of SH in controlling the signaling mediated by TLR-7 downstream effectors, the 293 cells were transfected with the IFN-α4 promoter linked to the luciferase reporter gene (a gift from Dr. Luke O’Neil, University of Dublin, Ireland) together with MyD88, IκB kinase (IKK)-α and TRAF6 or TRAF3 expression plasmids in the presence of SH expression plasmid or its empty vector, treated with recombinant IFN-α (100 μg/mL) to induce TLR7 expression [19], and harvested at 40 h post-transfection to measure luciferase activity.

### 2.5. Statistical Analysis

Analysis was performed with the InStat 3.05 Biostatistics Package (GraphPad, San Diego, CA, USA) using one-way ANOVA to determine differences among groups. Data are expressed as mean ± the standard error (SEM) values.

## 3. Results

### 3.1. The SH Protein Inhibits Type I IFN Secretion in pDCs 

**Figure 1:**
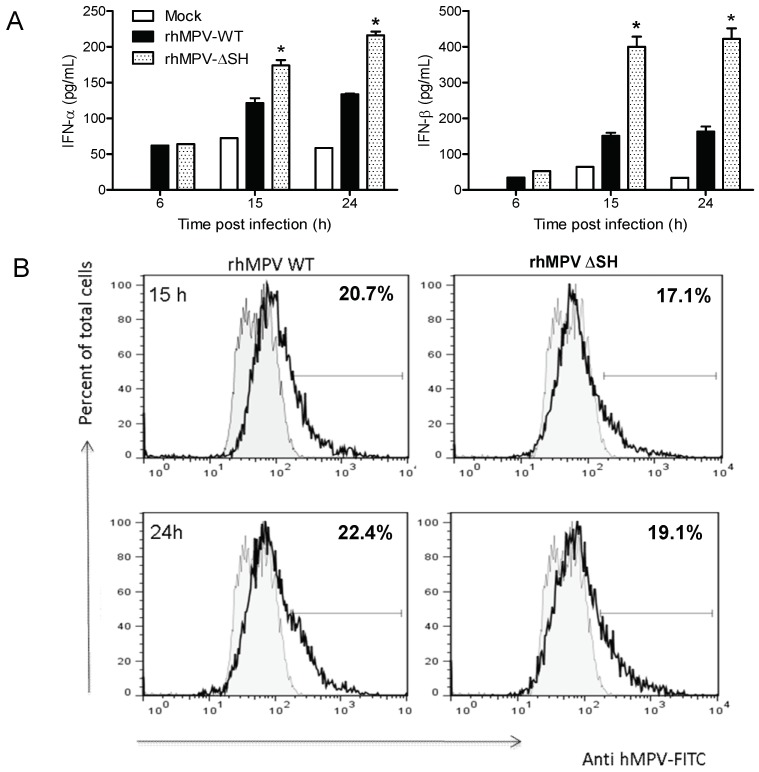
hMPV SH glycoprotein inhibits type I interferon production by human pDCs. (**A**) Isolated human pDCs (1 × 10^5^ in 200 μL complete medium in 96-well plate) were infected with rhMPV-WT or −∆SH at MOI of 3. At various time points p.i., cell free supernatants were harvested to measure IFN-α and -β secretion by ELISA. (**B**) Intracellular detection of viral antigens. Virus infected cells were first fixed, permeabilized, and incubated with guinea pig anti-hMPV antibody followed by a FITC-goat anti-guinea pig antibody (Zymed). Cells were analyzed with a FACScan flow cytometer equipped with CellQuest software 8 (San Jose, CA, USA). Analysis was performed in FlowJo software 10 (Treestar, CA, USA). Data represent the mean ± SEM of five independent experiments (*n* = 5 donors). * *p* < 0.05 relative to rhMPV-WT.

In an attempt to dissect the underlying mechanism of hMPV inhibition of IFN production, purified human pDCs were infected with either rhMPV-WT or rhMPV-ΔSH. Cells were harvested at different times post-infection (p.i.) to collect supernatants for subsequent measurement of type I IFN by ELISA. As shown in Figure 1A, rhMPV-ΔSH infection induced a significantly higher amount of both IFN-α and β from pDCs compared to rhMPV-WT, suggesting that the SH protein inhibits type I IFN production in these cells. To determine whether the enhanced production of IFN by rhMPV-ΔSH-infected cells was due to a difference in the level of viral replication, intracellular staining of hMPV was performed. There was no significant difference between cells infected with rhMPV-WT or rhMPV-ΔSH both at 15 and 24 h p.i., although there was a trend in the slightly reduced percentage of infected cells in the case of rhMPV-ΔSH infection (Figure 1B). 

### 3.2. TLR7 and MyD88 are Essential for the Induction of Type I IFN by hMPV 

Multiple studies have reported that pDCs respond to viruses and viral products primarily through the recognition of pathogen-associated molecular patterns by the two intracellular Toll-like receptors (TLR), TLR7 and TLR9, which detects single stranded RNA and unmethylated DNA motifs, respectively [20,21,22,23]. TLR signaling is initiated by the interaction between the cytoplasmic domain of TLR with TIR-domain-containing cytosolic adaptors. MyD88 is the common TIR-domain-containing adaptor of all TLRs except for TLR3 [23,24,25]. During viral infection, MyD88 recruits members of the interleukin-1 receptor-associated kinase (IRAK)-1 and/or -4 to activate transcription factors belonging to the NF-κB and activator protein 1 (AP-1) family via TRAF6 and transforming growth factor beta-activated kinase 1 (TAK1) [26,27,28]. The activation of IKKε/tank binding kinase 1 (TBK-1) in response to dsRNA, leading to IRF phosphorylation, is controlled by TIR-domain-containing adapter-inducing interferon-β (TRIF) association with TLR3 or TRIF related adaptor molecule (TRAM) [24].

**Figure 2:**
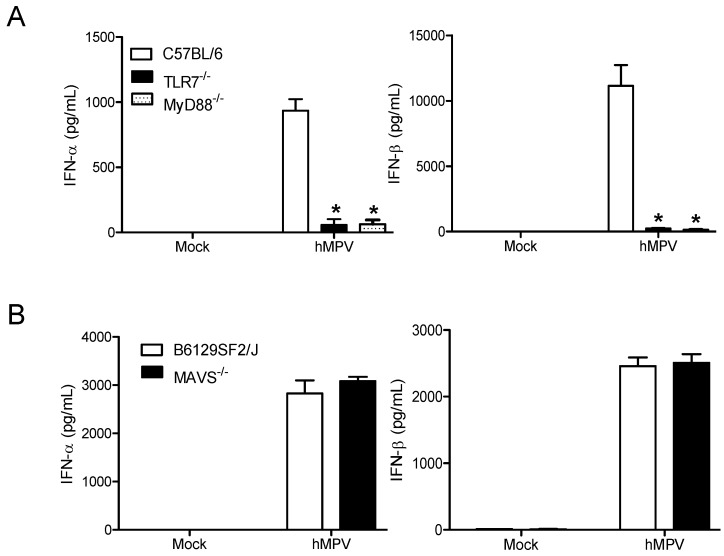
hMPV induced type I interferon in pDCs is TLR-7/MyD88 dependent. Spleen pDCs isolated from C57BL/6, TLR7-/- and MyD88-/- mice (**A**) or B6129SF2/J or MAVS-/- mice (**B**) were infected with hMPV (MOI 3) and supernatant was harvested at 24h p.i. to measure IFN-α and -β production by ELISA. Each bar represents mean ± SEM (*n* = 4 animals/group) and representative of three independent experiments. * *p* < 0.05 relative to wild type cells.

To confirm the role of TLR7 in hMPV-induced signaling in pDCs [29], spleen pDCS were isolated from TLR7-/- mice and interferon secretion was measured following hMPV infection. The lack of TLR7 completely blocked the production of hMPV-induced type I interferon (Figure 2A) compared to the wild type control mice (C57BL/6, Jackson). Similar results were obtained with pDCs isolated from MyD88-/- mice, provided by Dr. Akira from the Hyogo College of Medicine, Japan (Figure 2A). As we previously found that hMPV activates the retinoic acid-inducible gene I (RIG-I)-mitochondrial antiviral-signaling protein (MAVS) signaling pathway in airway epithelial cells, leading to the expression of proinflammatory and antiviral molecules [30], we also infected spleen pDCs isolated from MAVS-/- mice (Jackson) and their relative control mice. As expected, there was no difference in IFN secretion between MAVS-/- and control mice infected cells (Figure 2B), indicating that hMPV-induced IFN secretion in pDCs is mediated primarily via the TLR7/MyD88 pathway.

### 3.3. SH Suppresses TLR-7-Dependent Type I IFN Expression 

**Figure 3:**
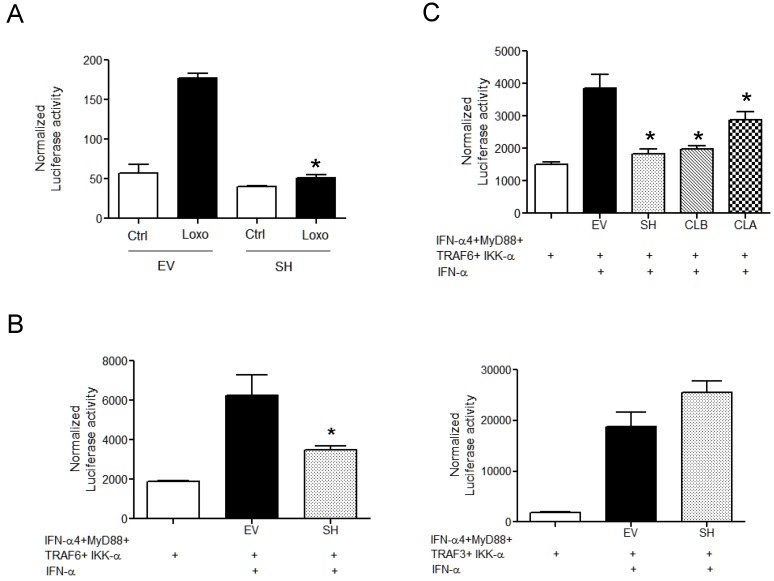
hMPV SH protein inhibits TLR7-dependent signaling. (**A**) 293 cells stably expressing TLR 7 were co-transfected with an IRF-driven promoter and either the SH expression plasmid (1 g) or its empty vector (EV) and stimulated with 1 mM loxorabine. Cells were lysed 40 h post-transfection to measure luciferase activity. (**B**) 293 cells were co-transfected with IFN-α4 promoter (0.1 g), linked to a luciferase reporter gene together with MyD88 (0.1 g), IKK-α (0.1 g), and TRAF6 (0.5 g) (**left panel**) or TRAF3 (0.5 g) (**right panel**) expression plasmids in the presence of SH expression plasmid (1 g), or its empty vector (EV). Cells were treated with recombinant IFN-α (100 μg/mL), to activate TLR7, and harvested 40 h later to measure luciferase activity. (**C**) 293 cells were co-transfected as described in panel B with TRAF6 and a plasmid expressing the SH protein isolated from a representative clinical isolate A (CLA) or B (CLB). Cells were harvested 40 h later to measure luciferase activity. Data are representative of three independent experiments run in triplicate and are expressed as means ± SEM of normalized luciferase activity to the β-galactosidase reporter activity. * *p* < 0.05 relative to the empty vector.

To better investigate the role of hMPV SH in modulating TLR7-dependent signaling, HEK293 cells stably expressing TLR7 and their wild type counterpart were transfected with an ISRE-driven luciferase reporter gene plasmid, together with a plasmid for SH expression, or its empty vector, and treated with the TLR7 ligand loroxibine. As shown in Figure 3A, expression of SH protein significantly inhibited loxoribine-induced IRF-driven reporter activity. 

The engagement of TLR7 induces the complex formation of IRF7 with MyD88, interleukin-1 receptor-associated kinase 4 (IRAK4), TRAF6, TRAF3, and IKKα. To better investigate the ability of SH protein to inhibit TLR7-dependent IFN gene transcription, 293 cells were transfected with the IFN-α4 promoter controlling luciferase reporter plasmid, together with plasmids expressing MyD88, IKK-α, and TRAF6 or TRAF3 in the presence/absence of SH expression plasmid, followed by recombinant IFN-α treatment to induce TLR7 expression [19]. As shown in Figure 3B, SH protein expression resulted in the inhibition of TRAF6-, but not TRAF3-dependent IFN-α4 promoter activation (Figure 3B), indicating that SH targets TRAF6 to inhibit TLR7-induced IFN secretion.

To determine whether the SH inhibitory function was conserved between A and B strains, the SH protein was cloned from a representative A and B clinical isolate (a gift from Dr. Pedro Piedra, Baylor College of Medicine and Dr. John Williams, University of Pittsburgh, Pittsburgh, PA, USA, respectively) and expressed in 293 cells together with the IFN-α4 promoter reporter plasmid, MyD88, IKK-α, and TRAF6. All SH proteins demonstrated a similar ability to inhibit TRAF6-dependent IFN-α4 promoter activation (Figure 3C).

## 4. Discussion

The role of hMPV SH protein in host immunity was first described in non-immune cells. We found that SH suppresses hMPV-induced NF-κB activation in lung epithelial cells [6]. hMPV SH protein also downregulates type I IFN signaling by affecting STAT1 expression and phosphorylation in the infected lung epithelial cells [31]. A similar immune regulatory role was also demonstrated for the SH protein of respiratory syncytial virus (RSV), a close family member of hMPV. IL-1β secretion in RSV-infected airway epithelial cells was higher than in cells infected with a recombinant hRSV, lacking the SH protein [32,33], suggesting the importance of SH from *Pneumoviridae* family in host innate immunity. However, whether hMPV SH protein plays such a role in immune cells is largely unknown. 

Upon the entry of bacterial and viral pathogens, pDCs represent an important source to produce IFN for host defense [10,11,12]. We have recently shown that hMPV can infect human pDCs and induce a significant amount of type I IFN [13]. Although the TLR7/9/MyD88/TRAF6 pathway has been proposed to be responsible for hMPV-induced IFN-α in pDCs, the experiments were not directly performed in pDCs, but in 293 cells [34]. Herein, we used pDCs from TLR7-/-, MyD88-/-, and MAVS-/- mice and identified that type I IFN induction by hMPV depends on the TLR7/MyD88 pathway. MAVS, a significant host factor responsible for the type I IFN in non-immune cells, does not play a role in type I IFN induction in pDCs, demonstrating that the IFN induction mechanisms are cell type-dependent. 

In response to host immunity, viruses always develop immune evasion strategies against host defense. We have shown that hMPV infection inhibits TLR-dependent signaling in pDCs [13], similar to what has been described for respiratory syncytial virus and measles virus [14,15]. Herein, we further demonstrated a mechanism contributing to the suppression. We found that hMPV uses the SH protein to inhibit type I IFN induction in pDC via interfering with the TLR7/MyD88/TRAF6 pathway. Similar roles have been found for paramyxovirus C protein, which suppresses IFN-α induction by blocking TLR7/9-mediated pathway in pDC [35], and parainfluenza type 2 V protein, which binds to TRAF6 and inhibits IRF7 lysine 63 polyubiquitination to affect type I IFN secretion [19]. 

In addition to the role in type I IFN induction in pDC and NF-kB activation and type I IFN signaling in airway epithelial cells [6,31], the hMPV SH protein has also been shown to inhibit micropinocytosis-mediated entry into human dendritic cells and reduce CD4 T cell activation [36]. All these findings support the knowledge that SH is a significant regulator for hosts’ innate immune response to hMPV infection.
